# Two *Drosophila *suppressors of cytokine signaling (SOCS) differentially regulate JAK and EGFR pathway activities

**DOI:** 10.1186/1471-2121-5-38

**Published:** 2004-10-15

**Authors:** Jason S Rawlings, Gabriela Rennebeck, Susan MW Harrison, Rongwen Xi, Douglas A Harrison

**Affiliations:** 1 Dept. of Biology, University of Kentucky, 101 Morgan Bldg. Lexington, KY, 40506, USA

## Abstract

**Background:**

The Janus kinase (JAK) cascade is an essential and well-conserved pathway required to transduce signals for a variety of ligands in both vertebrates and invertebrates. While activation of the pathway is essential to many processes, mutations from mammals and *Drosophila *demonstrate that regulation is also critical. The SOCS (Suppressor Of Cytokine Signaling) proteins in mammals are regulators of the JAK pathway that participate in a negative feedback loop, as they are transcriptionally activated by JAK signaling. Examination of one *Drosophila *SOCS homologue, Socs36E, demonstrated that its expression is responsive to JAK pathway activity and it is capable of downregulating JAK signaling, similar to the well characterized mammalian SOCS.

**Results:**

Based on sequence analysis of the *Drosophila *genome, there are three identifiable SOCS homologues in flies. All three are most similar to mammalian SOCS that have not been extensively characterized: Socs36E is most similar to mammalian SOCS5, while Socs44A and Socs16D are most similar to mammalian SOCS6 and 7. Although Socs44A is capable of repressing JAK activity in some tissues, its expression is not regulated by the pathway. Furthermore, Socs44A can enhance the activity of the EGFR/MAPK signaling cascade, in contrast to Socs36E.

**Conclusions:**

Two *Drosophila *SOCS proteins have some overlapping and some distinct capabilities. While Socs36E behaves similarly to the canonical vertebrate SOCS, Socs44A is not part of a JAK pathway negative feedback loop. Nonetheless, both SOCS regulate JAK and EGFR signaling pathways, albeit differently. The non-canonical properties of Socs44A may be representative of the class of less characterized vertebrate SOCS with which it shares greatest similarity.

## Background

The vertebrate JAK signaling pathway is an essential component of cellular response to a wide array of cytokines and growth factors. The JAK cascade is reutilized for signaling events in numerous tissues and at multiple stages of mammalian development [reviewed by [1-3]]. Many interleukins, interferons, and growth factors are among the ligands that stimulate signaling through the JAK pathway. The pathway can also be stimulated through activation of some receptor tyrosine kinases, including epidermal growth factor receptor (EGFR). As a result of its broad utilization, JAK signaling is essential for many developmental events.

Though the JAK pathway is vital to many developmental processes, strict control of JAK signaling is equally important. As with other signaling pathways, mechanisms must be in place to balance the activation of JAK pathway activity. Regulation serves to "reset" the pathway so that it will be responsive to subsequent signals and it restricts the level or duration of the signal so that it is properly interpreted by the cell. Inappropriate JAK activation is the direct cause of a specific form of acute lymphocytic leukemia (ALL) [4-6]. In addition, JAK/STAT activation has been strongly correlated with a variety of cancers, including many blood cell and immune cell transformations [reviewed by [7-9]]. Furthermore, in cell culture, constitutive activation of c-Eyk, v-src, or v-abl results in the constitutive activation of specific STATs or JAKs [10-13]. These examples highlight the necessity of regulating JAK/STAT activation.

Because of the need to limit JAK activity, it is not surprising that there are several conserved protein families that regulate JAK activation [reviewed by [3,14,15]]. These include phosphatases, Protein Inhibitors of Activated STATs (PIAS), and, the best characterized, the suppressors of cytokine signaling (SOCS) family. In mammals, eight different SOCS genes have been found [16]. These SOCS proteins have a distinctive modular architecture: a central SH2 domain followed by a carboxyl terminal SOCS domain, while the amino termini are quite divergent. Biochemical investigations have revealed that SOCS proteins use multiple mechanisms to regulate activity of the JAK pathway [see reviews, [3,9]]. First, the SOCS SH2 domain can bind to the phosphorylated receptor, thereby prohibiting access to positive effectors of the pathway. Second, at least some SOCS can specifically inhibit the catalytic activity of JAKs. Lastly, SOCS binding to activated JAK pathway components may target those proteins for degradation. The SOCS motif interacts with the elongins B and C, which bind to cullins and are E3 ubiquitin ligases [17,18]. Addition of ubiquitin to the bound proteins would target them for proteasomal degradation. Therefore, the negative influence of SOCS on its substrates may be due to multiple distinct mechanisms.

Use of the JAK signaling pathway for developmental processes is not restricted to mammals. Indeed, the JAK cascade is evolutionarily conserved, and can be found as an intact signaling pathway even in insects [3,19-21]. In *Drosophila*, the JAK pathway is involved in embryonic patterning, sex determination, blood cell development, patterning of adult structures, planar polarity of photoreceptor clusters, maintenance of stem cells in spermatogenesis, and follicle cell patterning and function [see reviews [19,21]]. Furthermore, the fly JAK pathway must also be properly regulated to avoid deleterious effects. As in vertebrates, hyperactive JAK signaling has also been shown to directly cause neoplastic cell growth in *Drosophila*. Two dominant gain-of-function alleles of *hopscotch *result in hypertrophy of the larval lymph glands, the hematopoietic organ, and melanotic masses [22-24]. Excess activity in the blood system causes overproliferation and differentiation of the macrophage-like blood cells, creating leukemia-like effects. Inappropriate activity in the developing tissues of the adult fly can also cause alteration of the development of the adult thorax, wing veins, head, eyes, and ovaries [22,25-27].

Of the eight mammalian SOCS, four have been studied extensively (CIS, SOCS1-3). These genes have been shown to respond to JAK pathway activation and subsequently are able to downregulate its activity as described above, completing a classical negative feedback loop. In comparison, very little is known of the remaining four. Here we present the identification and characterization of *Drosophila *Socs44A. It contains the same modular domain architecture as mammalian SOCS and shows greatest sequence similarity to the relatively uncharacterized SOCS6 and SOCS7. We show that, unlike the previously studied *Drosophila Socs36E *[28,29], *Socs44A *expression in embryogenesis is independent of JAK pathway activity. However, Socs44A is able to regulate the JAK cascade in embryogenesis, but not in oogenesis. Finally, *Socs44A *genetically interacts with and upregulates the EGFR/MAPK pathway. The characteristics of Socs44A that distinguish it from the canonical Socs36E may be representative of features that are shared with the class of less-defined mammalian SOCS genes.

## Results

### The *Drosophila *genome encodes three putative SOCS genes

**Figure 1:**
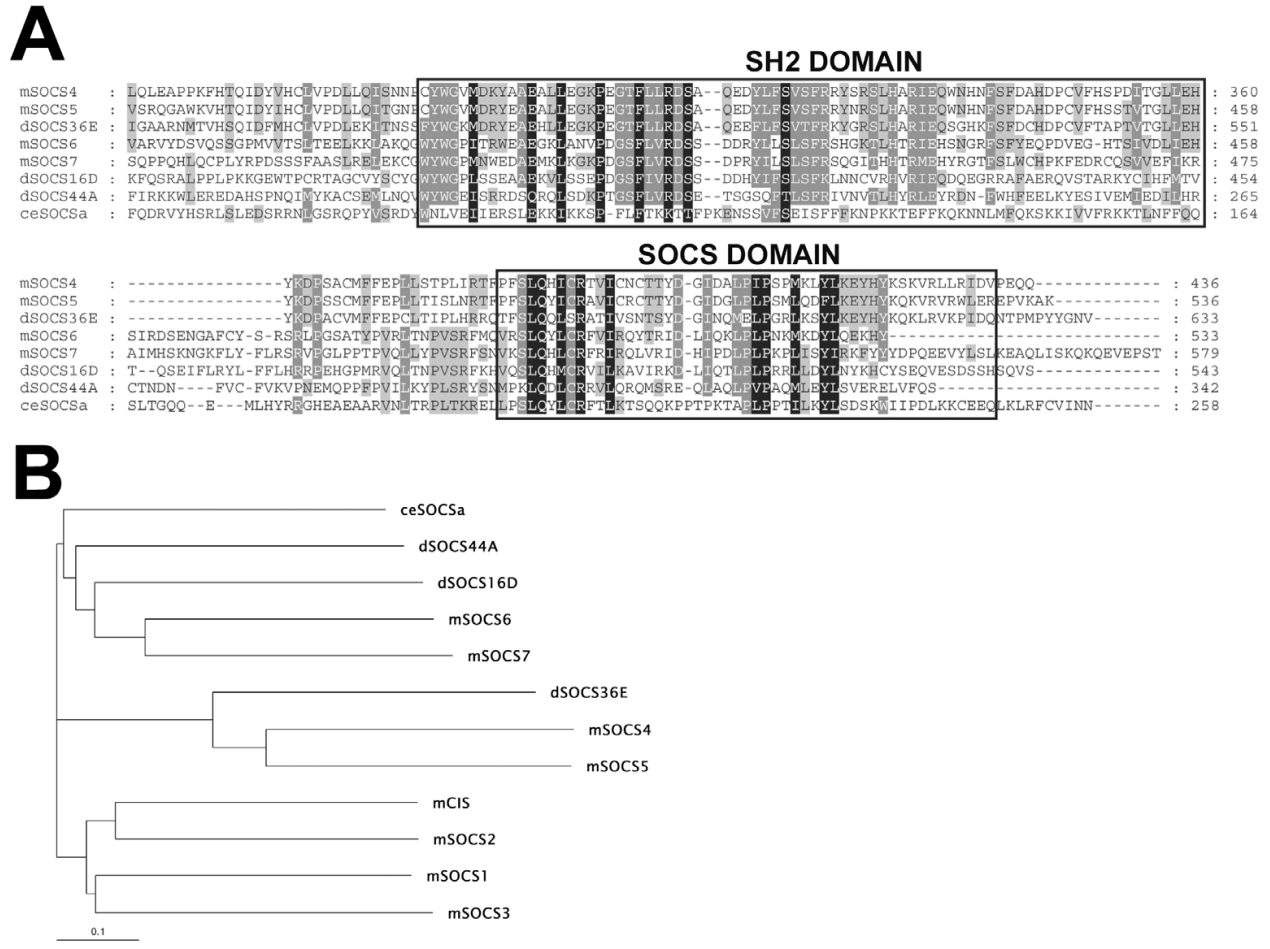
**Protein sequence comparison of *Drosophila *and mouse SOCS. **(A) The predicted carboxyl terminal protein sequences of *Drosophila *(d), mouse (m), and *C. elegans *(ce) SOCS genes, including the SH2 and SOCS box domains, are aligned and shaded to indicate similarities and identities. (B) Based on the protein alignments, the neighbor-joining method was used to construct a phylogenetic tree of these SOCS.

Based on the consensus protein sequence for a SOCS box derived by Hilton and colleagues [30], a tBLASTn search of the Berkeley *Drosophila *Genome Project (BDGP) database [31] was conducted to examine all possible reading frames. Three putative loci containing both a SOCS box and an SH2 domain were identified using this strategy. All three match the arrangement of mammalian SOCS genes in that the SOCS box is at the carboxyl terminus with the SH2 domain directly preceding it. Each of these putative homologues also overlaps with a predicted gene from the BDGP. We named these three genes *Socs16D *(overlapping with CG8146), *Socs36E *(overlapping with CG15154), and *Socs44A *(CG2160), based upon their cytological location. Comparison of these three fly SOCS genes with vertebrate SOCS reveals that Socs36E is most similar to the mouse SOCS5, while Socs16D and Socs44A are less similar to specific mouse SOCS (see Fig. 1). While the amino termini are quite different, SOCS5 and Socs36E are 62% identical (71% similar) at the carboxy terminus from the region just before the SH2 domain to the end of the SOCS domain (region shown in Fig. 1A). Within that same C-terminal region, Socs44A is most similar to SOCS6 and SOCS7 (46% and 39% similar, respectively). Socs16D also has highest similarity to SOCS6 and SOCS7 (47% to each) over the same carboxyl region. These similarities suggest that the ancestral versions of *Socs36E *and a common predecessor of *Socs16D *and *Socs44A *existed as two separate SOCS genes at the time of divergence of mammals and dipterans (Fig. 1B).

### Socs44A expression is not regulated by JAK pathway activity

**Figure 2:**
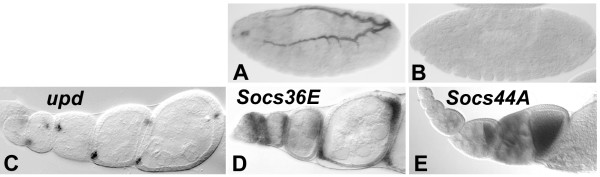
***Socs36E *and *Socs44A *are expressed in different spatio-temporal patterns. **The embryonic expression patterns of *upd *and *Socs36E *are dynamic from early blastoderm throughout embryogenesis [see 28, 29 and Fig. 3]. *Socs44A *expression is not detected until very late stages in the trachea (A). Although such staining can be artifactual, sense strand probe never showed any staining (B). In the ovary, *upd *is expressed specifically in the polar follicle cells at each end of the chamber (C). *Socs36E *expression encompasses the anterior and posterior follicular epithelium, with highest expression at the poles (D). This is consistent with activation of *Socs36E *transcription due to reception of the Upd ligand which is secreted from the polar follicle cells and diffuses toward surrounding cells. *Socs44A *expression is restricted to the germline and only during later stages of oogenesis (E)

**Figure 3:**
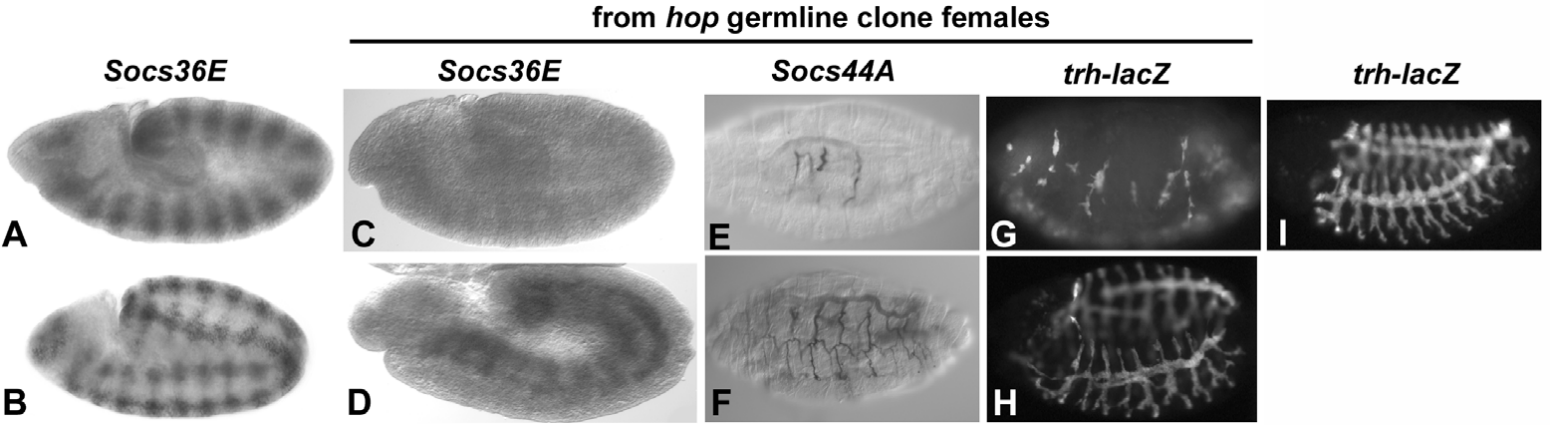
**Loss of JAK activity does not affect *Socs44A *expression. **As compared with wild-type at various embryonic stages (A and B), germline clone derived embryos from *hop*^*c111 *^mothers (C-H) display dramatically reduced or eliminated expression of *Socs36E *(C and D). Only a stripe of mesodermal staining in germ band extended embryos (D) remains at nearly normal intensity in the mutant embryos. In contrast, expression of *Socs44A *in trachea persists in *hop*^*c111 *^germline clone-derived embryos that are unrescued (E) or paternally rescued (F). However, the trachea are morphologically altered and drastically reduced in unrescued (G) and paternally rescued (H) animals, as compared with wild-type (I), as evidenced by a *trachealess *enhancer trap (G-I).

In mammals, regulation of JAK signaling through SOCS proteins is based on a simple negative feedback mechanism. Specifically, the activity of the JAK pathway stimulates the expression of SOCS genes, because activated STATs bind to enhancers for the SOCS genes and induce transcription. *Socs36E *is similarly regulated during embryogenesis by *Drosophila *JAK signaling [29]. *Socs36E *is expressed dynamically, in a striped pattern that later becomes restricted predominantly to the tracheal pits [[28,29], and Fig. 3], very similar to *upd*, the gene encoding the embryonic ligand for the JAK pathway [32]. Indeed, activation of JAK signaling is both necessary and sufficient for *Socs36E *expression in embryogenesis [29]. Furthermore, the expression of Socs36E during oogenesis matches the known activation of JAK signaling. The expression of *upd *in the ovaries is restricted to the two polar follicle cells at either end of the egg chambers of the vitellarium [[26] and Fig. 2C]. *Socs36E *is expressed in a larger number of follicle cells centered at the two poles of the egg chamber (Fig. 2D). Given that secreted Upd protein is produced in the polar follicle cells and activates JAK signaling in neighboring cells [33], this suggests that *Socs36E *expression is controlled by JAK activity in oogenesis, as well as embryogenesis.

The *Socs44A *gene that was predicted based on protein homology is identical to hypothetical gene CG2160. A single cDNA corresponding to the locus (LP02169) was isolated by the BDGP, has been completely sequenced and encodes the expected SH2 and SOCS domains at the carboxyl terminus (gb AF435923). To determine whether *Socs44A *is similarly regulated by JAK pathway activity, in situ hybridization to embryos and ovaries was performed. No specific expression of *Socs44A *was detected until very late in embryogenesis. The only striking staining pattern observed was in the trachea of late embryos (Fig. 2A). Non-specific tracheal staining is sometimes seen with probes to late embryos, however this pattern was never observed when sense probe was used (Fig. 2B). Unfortunately, embryos homozygous for any available deletions that remove Socs44A die prior to formation of trachea, therefore we cannot conclusively determine whether the late tracheal staining reflects RNA expression. Nonetheless, because the JAK pathway is activated in a segmentally repeated pattern during embryogenesis, the lack of *Socs44A *expression suggests that it is not responsive to JAK signaling. Consistent with this conclusion, expression of *Socs44A *in the ovary is restricted to only germline expression late in oogenesis, with no detectable RNA in the follicular epithelium (Fig. 2E).

To directly test whether *Socs44A *expression is regulated by JAK pathway activity, in situ hybridization to *Socs44A *RNA was performed in embryos that lack JAK pathway activity. The product of the *hop *gene is required in early embryogenesis and must be provided maternally for proper segmentation of the embryo. The dominant female sterile (DFS) technique was used to generate females that fail to produce *hop *in the germline [34]. In situ hybridization of *hop *germline clone embryos using *Socs36E *as probe demonstrates a strong reduction in *Socs36E *expression in the mutant embryos as compared with wild-type (Fig. 3). Similar results have been reported in embryos lacking *upd *activity [29]. These data demonstrate that *hop *is required to stimulate the normal segmentally-repeated *Socs36E *expression in the embryo. However, expression of *Socs44A *does not appear to be affected by maternal loss of *hop*. Although the trachea are malformed and dramatically reduced in embryos lacking JAK pathway activity [[35,36] and Fig. 3G,3H], the remaining segments of trachea continue to express *Socs44A *at apparently normal levels (Fig. 3E,3F). Thus the failure of endogenous *Socs44A *to be expressed in the normal pattern of JAK pathway activation and of *Socs44A *expression to be eliminated by loss of JAK activity indicate that *Socs44A *expression is not stimulated by the pathway.

Activity of the JAK pathway is both necessary and sufficient for the expression of *Socs36E*. The ectopic activation of the JAK pathway by misexpression of *upd *results in expression of *Socs36E *in the same pattern [[29] and data not shown]. In contrast, similar misexpression of UAS-upd with the paired-GAL4 driver failed to stimulate any detectable expression of *Socs44A *in the embryo (not shown). We conclude that *Socs44A *expression is not responsive to JAK pathway activity, therefore cannot function via a traditional auto-regulatory feedback loop.

### Ectopic SOCS activity suppresses JAK signaling in the wing

**Figure 4:**
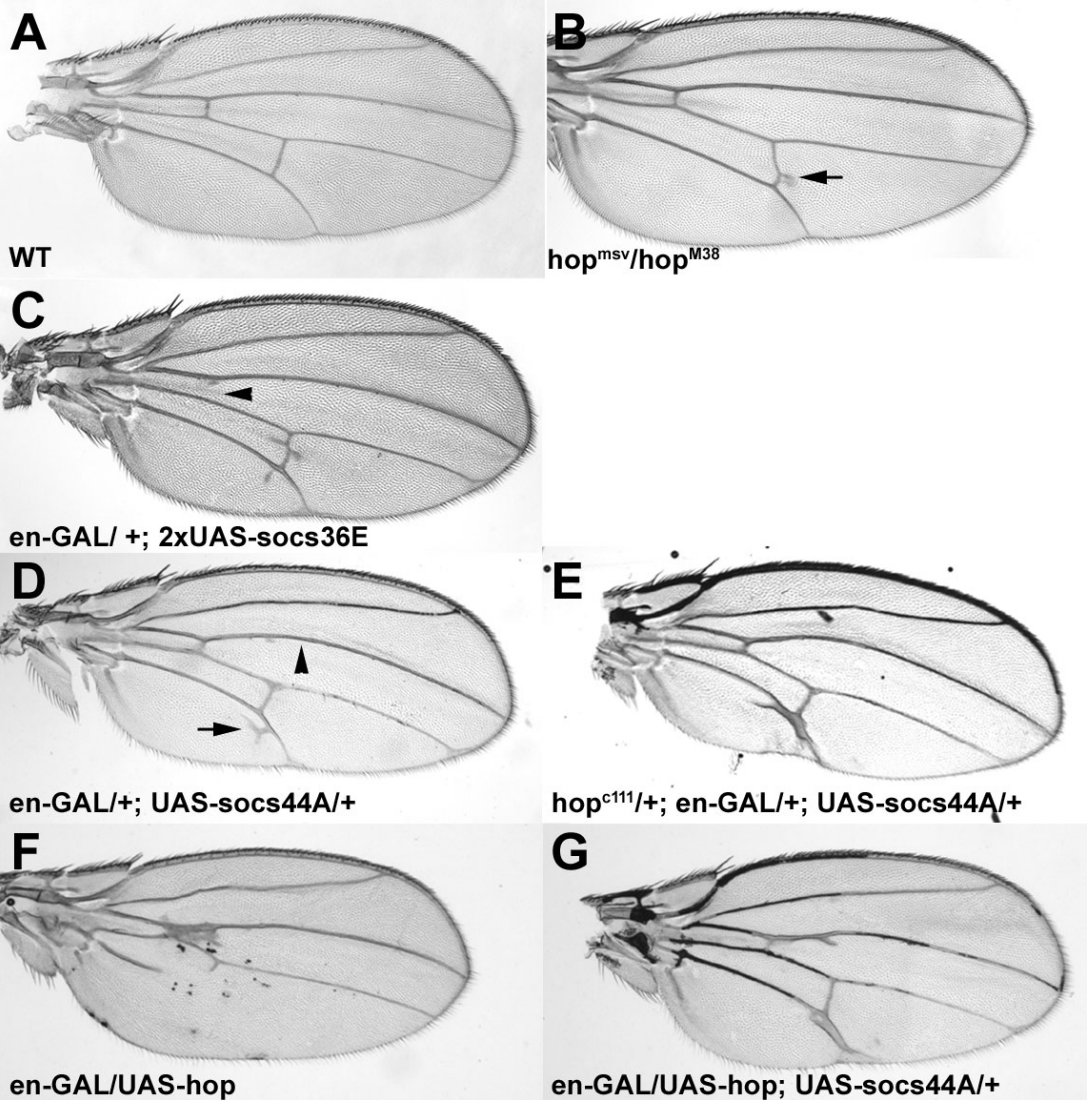
***Socs44A *misexpression reduces JAK signaling in the wing. **Wild-type venation (A) is compared with a viable *hop *mutant, *hop*^*msv*^/*hop*^*M38 *^(B). *hop *reduction causes ectopic vein (arrow) near the posterior crossvein. (C) Expression of UAS-Socs36E using the engrailed-GAL4 driver (e16E-GAL) produces a similar ectopic vein phenotype, plus the loss of the anterior crossvein (arrowhead). (D) Similar misexpression of *Socs44A *causes ectopic wing vein production near the posterior crossvein (arrow) and arching of vein L3 (arrowhead). (E) Reduction of the dosage of *hop *enhances the *Socs44A *misexpression phenotype. (F) Misexpression of *hop *in the posterior compartment causes dramatic vein loss, but that loss is restored by the simultaneous expression of *Socs44A *(G).

The lack of transcriptional regulation by JAK signaling does not preclude a role for Socs44A in the control of JAK activity. To test whether it can attenuate JAK signaling, *Socs44A *was misexpressed using the GAL4/UAS system. Similar experiments performed with *Socs36E *have demonstrated that expression in the developing wing reproducibly results in the production of ectopic wing vein near the posterior crossvein [Fig. 4C and [28]]. This phenotype is quite similar to that noted for viable mutants of *hop *or *Stat92E *[Fig. 4B and [37]], suggesting that *Socs36E *misexpression may cause a reduction in JAK signaling in the wing. But unlike observed JAK mutations, the anterior crossvein was also completely missing from *Socs36E *misexpression wings, perhaps suggesting an additional role for Socs36E that is independent of the JAK pathway. Callus and Mathey-Prevot [28] demonstrated that the additional influence on wing venation may be due to the suppression of the EGFR pathway.

Using the engrailed-GAL driver, GAL-e16E, expression of *Socs44A *in the posterior compartment of the wing caused mild venation defects similar, but not identical, to *Socs36E *(Fig. 4D). Expression of *Socs44A *caused production of ectopic wing vein near the posterior crossvein, but unlike *Socs36E*, the ectopic vein was seen predominantly posterior to L5, not between L4 and L5. Furthermore, the anterior crossvein was not reduced or eliminated by *Socs44A *expression, but a substantial arching of L3 was noticed. Both the ectopic vein and arching of L3 were enhanced in animals heterozygous for a null allele of *hop *(Fig. 4E), indicating that the phenotype is sensitive to a reduction in JAK pathway activity. Misexpression of *hop *activates JAK signaling and causes reduction of wing venation in the posterior of the wing, somewhat the opposite of *Socs44A *misexpression (Fig. 4F). The simultaneous misexpression of *hop *and *Socs44A *results in a phenotype similar to expression of *Socs44A *alone (Fig. 4G). Therefore, the activity of Socs44A is capable of negating the influence of ectopic JAK activity in the wing.

Loss of JAK function in embryos is lethal, but various combinations of weak alleles of *hop *show some viability (Table 1). If Socs44A were negatively regulating the JAK pathway, misexpression of *Socs44A *in a *hop *mutant background would be expected to further reduce viability. The ability of *Socs44A *misexpression to enhance the lethality of weak heteroallelic combinations of *hop *was tested. For all alleles examined, expression of *Socs44A *in the *engrailed *pattern caused complete lethality. For the weakest *hop *allelic combination, *hop*^*msv*^/*hop*^*M75*^, misexpression of *Socs44A *caused viability to drop from 62% to 0% (Table 1). These data are consistent with the hypothesis that ectopic *Socs44A *acts to further reduce pathway activity in these JAK activity depleted animals, causing lethality.

**Table 1:** Misexpression of Socs44A exacerbates the reduced viability of *hop *heteroallic mutants.

**Genotype**	**hop^M38 ^(n = 213)**	**hop^GA32 ^(n = 332)**	**hop^M75 ^(n = 172)**
**A**- hop^x^/FM7; en-GAL; TM3	33	52	21
**B**- hop^x^/FM7; en-GAL; UAS-socs44A	25	33	28
**C**- hop^x^/hop^msv^; en-GAL; TM3	11	20	13
**D**- hop^x^/hop^msv^; en-GAL; UAS-socs44A	0 (E = 8.33)	0 (E = 12.69)	0 (E = 17.33)

Misexpression of *Socs44A *in a range of *hop *heteroallelic mutants resulted in lethality. For each mutant combination, **x **is the allele of *hop *designated in the column heading, **n **represents the total number of progeny scored in the cross. **E **represents the expected number of progeny of that genotype if *Socs44A *misexpression were to have no effect on viability. The expected value is calculated using the formula **A/B=C/D**, which takes into account the change in viability imparted by homozygosity for *hop *relative to heterozygosity and the change in viability for misexpression of *Socs44A *relative to the GAL4 alone. The progeny scored here are derived from the cross: *hop*^*X*^/*Y Dp(1;Y)v*^+^*y*^+^*hop*^+^; *en-GAL4/CyO *males mated to *hop*^*msv*^/*FM7; UAS-socs44A/TM3 *females.

While the above data indicate that ectopic Socs44A is capable of downregulating JAK activity, they do not address whether Socs44A has an endogenous role in JAK pathway regulation. To determine if endogenous Socs44A downregulates JAK activity, we assayed the effect of a *Socs44A *deficiency on *hop *mutant phenotypes. The *hop*^*M38*/*msv *^heteroallelic mutant exhibits wing vein material at the posterior crossvein (Fig 4B) that is 98% penetrant. Removal of a single copy of *Socs44A *using either of two deficiencies in the region reduced the penetrance of the *hop *phenotype by as much as 52% (Table 2). An overlapping deficiency that did not remove the *Socs44A *locus had little effect on penetrance of the phenotype. These results suggest that regulation of JAK activity in the wing is a normal endogenous function of Socs44A.

**Table 2:** Endogenous Socs44A regulates JAK pathway activity.

	**+/+**	**CA53/+ (n = 237)**	**NCX10/+ (n = 292)**	**Drl/+ (n = 242)**
hop^M38^/hop^msv^	98% (of 89)	46% (of 13)	58% (of 12)	87% (of 15)

*hop*^*msv*^/*hop*^*M38 *^heteroallelic females have a wing spur phenotype (Fig. 4B) that is 98% penetrant (n = 89). The penetrance of the spur phenotype is dramatically reduced by removal of one copy of *Socs44A*, as seen for heterozygotes of *Df(2)CA53 *(CA53)and *Df(2)NCX10 *(NCX10). Rescue of the phenotype was not seen with *Df(2)Drl*^*rv18 *^(Drl), an overlapping deficiency that does not include *Socs44A*. Total number of animals is indicated by **n**, and number of animals of the indicated genotype is in parentheses.

### Socs44A upregulates EGFR pathway activity

**Figure 5:**
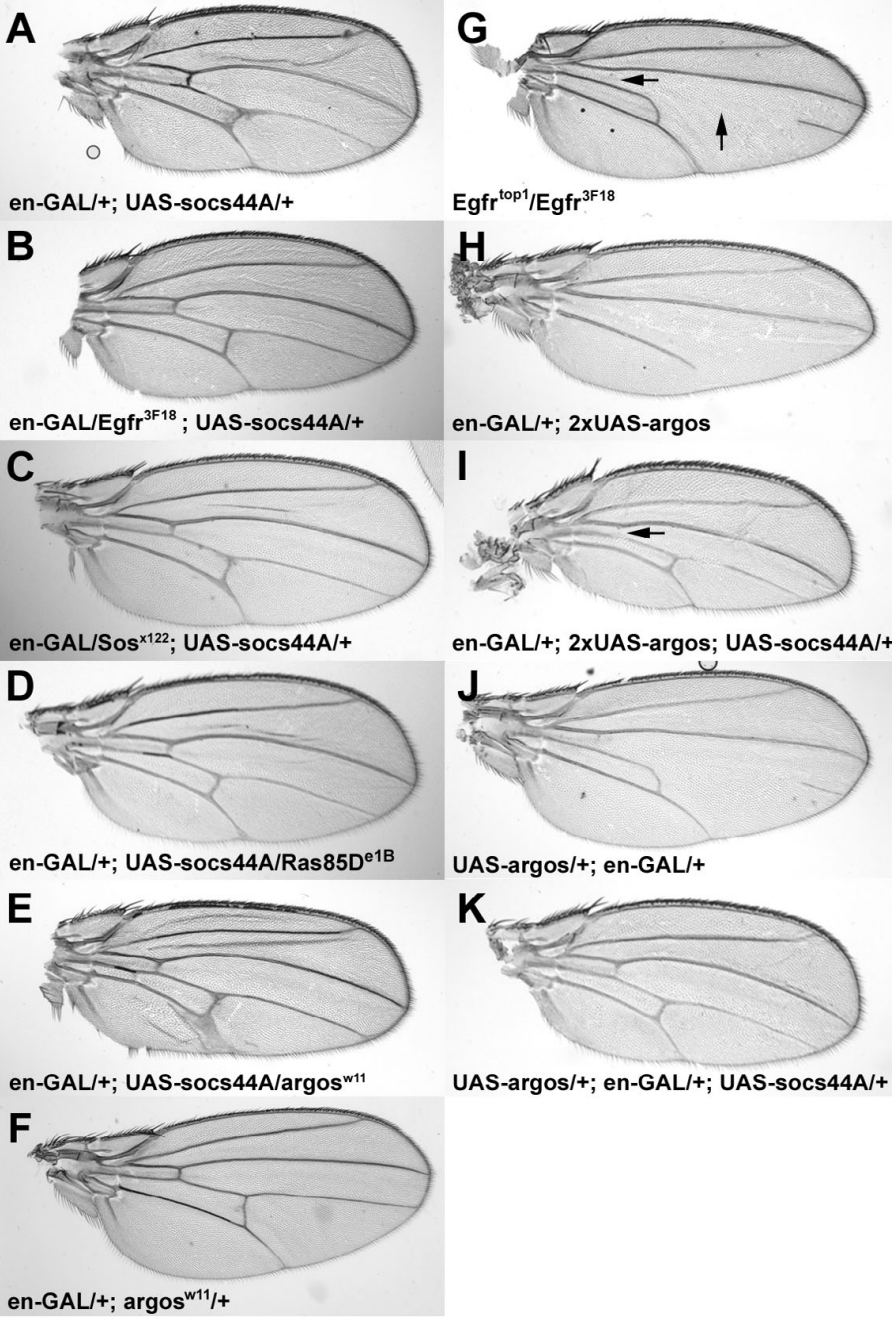
**Socs44A increases activity of EGFR signaling. **The ectopic wing vein phenotype of *Socs44A *misexpression (A) is rescued by reduction of *Egfr *(B), *Sos *(C) or *Ras85D *(D), positive effectors of EGFR signaling. In contrast, reduction of *argos*, a negative regulator of EGFR signaling, enhances the *Socs44A *misexpression phenotype (E). The *argos *allele combined with en-GAL have no effect on venation without the UAS-Socs44A transgene (F). Certain heteroallelic *Egfr *mutants possess a distinct wing vein phenotype, whereby the anterior crossvein and the central portion of L4 is missing (G, arrows). *Engrailed*-driven misexpression of *argos *has a similar phenotype (H and J). Concurrent misexpression of *Socs44A *antagonizes *argos *misexpression to restore near normal wing venation (I and K). The designation "2xUAS-argos" refers to presence of 2 total copies of the transgene in the genome.

In mammals, there are multiple points of cross-talk between the JAK and EGFR/MAPK signaling pathways [3,38-40]. EGFR signaling plays a prominent role in many developmental processes in *Drosophila*, including wing venation [41,42]. As mentioned above, expression of *Socs36E *has been reported to suppress EGFR signaling in the wings [28]. To determine the relationship of Socs44A to EGFR/MAPK signaling, wing phenotypes due to misexpression of *Socs44A *were examined in the background of heterozygous mutations for components of the EGFR signaling pathway. *Engrailed-GAL4 *driven misexpression phenotypes of Socs44A were suppressed in the background of heterozygous mutations for *Ras85D, Son of sevenless (Sos), *and *Egfr *(Fig. 5A,5B,5C,5D). Consistent with these observations, reduction in the dosage of the EGFR negative regulator *argos *enhanced the *Socs44A *misexpression phenotype (Fig. 5E). In contrast, concurrent misexpression of *Socs44A *and *argos *had antagonistic effects. Misexpression of two copies of an *argos *transgene under the *engrailed-GAL4 *driver resulted in wings lacking the 4^th ^lateral vein (L4) as well as both cross-veins (Fig. 5H). Concurrent misexpression of a single copy of the *Socs44A *transgene in this background was able to rescue this phenotype, restoring the posterior crossvein and both the most proximal and distal portions of L4 (Fig. 5I). The resulting wing phenotype mimicked that seen when only a single copy of *argos *was used in the misexpression assay (Fig. 5J) or what is seen in heteroallelic *Egfr *mutants (Fig. 5G). Finally, concurrent misexpression of a single copy of the *argos *and *Socs44A *transgenes produced a nearly wildtype wing (Fig. 5K). These data indicate that *Socs44A *expression is able to suppress *argos *misexpression phenotypes in a dose-dependent manner. It should be noted that concurrent misexpression of UAS-GFP did not affect the UAS-argos phenotype (not shown), indicating that the suppression by UAS-Socs44A was not merely a consequence of titrating GAL4.

**Figure 6:**
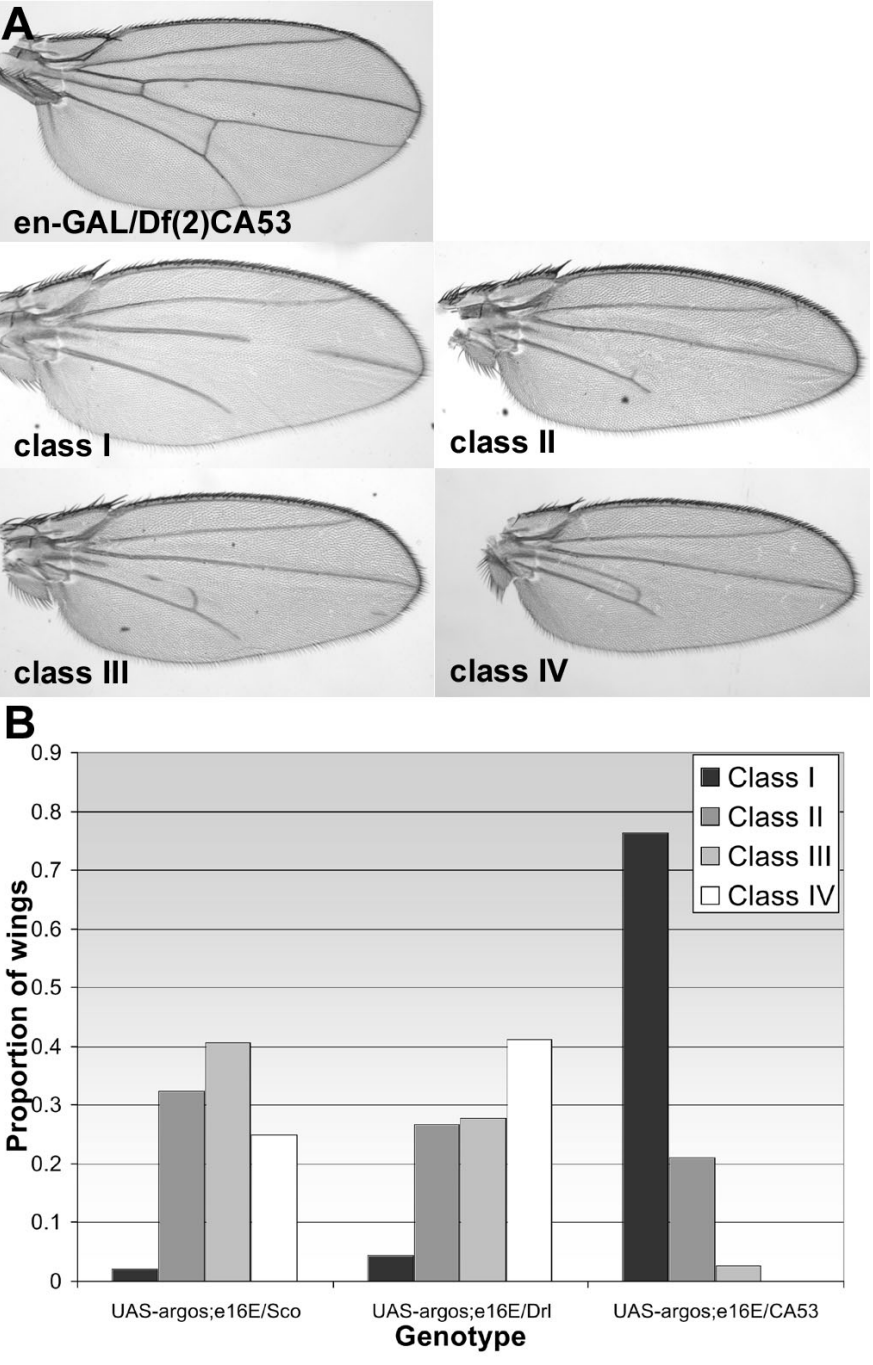
**Socs44A deficiencies enhance *argos *misexpression phenotypes. **(A) The *engrailed-GAL4 *driven misexpression of *argos *produces a range of phenotypes which were classified based on severity. The combination of en-GAL and *Df(2)CA53 *had no effect on venation. (B) In flies that were also heterozygous for *Df(2)CA53*, which removes the *Socs44A *locus, the distribution of phenotypes was significantly shifted to more severe classes as compared to animals heterozygous for *Df(2)Drl*^*rv18*^, an overlapping deficiency that does not remove *Socs44A *or for *Sco*, a chromosome wild-type for the 44A region.

Although these misexpression data indicate that Socs44A can enhance EGFR signaling, they do not necessarily demonstrate that this is a normal function of Socs44A. To address whether this is an endogenous function of Socs44A, we assayed the influence of a deficiency that removes *Socs44A *in the *argos *misexpression background. *Engrailed-GAL4 *misexpression of *argos *produces a range of phenotypic classes in which parts or all of L4 and/or the posterior cross-vein are missing (Fig. 6A). Addition of a single copy of a deficiency that removes *Socs44A *shifted the distribution of phenotypes to the more severe classes (Fig. 6B). In contrast, addition of an overlapping deficiency that does not include the *Socs44A *locus did not show such a shift. While it cannot be unambiguously stated that this effect is due to loss of *Socs44A *specifically, these results are consistent with the misexpression analyses and suggest that *Socs44A *normally plays a role in enhancing EGFR signaling in the *Drosophila *wing.

### Socs36E and Socs44A have different effects on oogenesis

**Figure 7:**
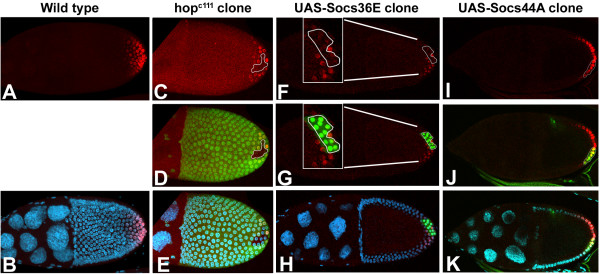
**Socs36E and Socs44A have different activities during oogenesis. **In wild-type ovaries (A, B), pnt-lacZ (red) is expressed in a gradient in the posterior terminal cells. Cells that lack *hop *activity (marked by a lack of green, see outline), also fail to express pnt-lacZ (C-E). Similarly, UAS-Socs36E misexpressed in clones (marked by presence of green, see outline), lack pnt-LacZ expression (F-H, see insets). In contrast, UAS-Socs44A misexpressed in clones (marked by presence of green, see outline), had no effect on pnt-LacZ expression (I-K). DAPI nuclear staining is shown in blue.

Evidence presented here and elsewhere indicates that Socs36E and Socs44A can downregulate JAK signaling in the wing [28]. However, the ability of specific mammalian SOCS to regulate JAK activity has been observed to differ, depending upon the tissue examined [43]. To determine whether there is a similar context specificity for the *Drosophila *SOCS, regulation was examined in another tissue in which JAK and EGFR functions have been well characterized. Both pathways are required for proper patterning of the follicular epithelium surrounding developing egg chambers during oogenesis [26,33,44-47]. One of the distinct cell populations requiring these pathways is the posterior terminal follicle cells [33]. These cells are molecularly identified by the expression of the ETS domain transcription factor, *pointed *[47-49]. In clones of cells that lack *hop *activity (Fig. 7C,7D,7E) or *egfr *activity (not shown), there is a loss of pnt-lacZ expression, indicating failure to specify the posterior terminal follicle cells.

To test whether Socs36E and Socs44A can downregulate JAK or EGFR activity during oogenesis, clones of cells misexpressing these genes in developing egg chambers were examined. In clones misexpressing *Socs36E *at high levels in posterior cells of the developing egg chamber, there was a dramatic loss of the pnt-LacZ marker (Fig. 7F,7G,7H). This loss was restricted to only those cells that misexpressed *Socs36E *and did not influence neighboring cells. These results indicate that JAK and/or EGFR signaling was attenuated by Socs36E activity. In contrast, for cells in which *Socs44A *was misexpressed in a similar fashion, there was no reduction of pnt-LacZ expression (Fig. 7I,7J,7K). We conclude that Socs44A is unable to attenuate JAK activity in the follicle cells. This ability of Socs44A to regulate JAK signaling in the wing, but not in the ovary, indicates that SOCS activity in invertebrates can also be context specific. Furthermore, the differential ability of the fly SOCS to attenuate JAK and EGFR signaling in the ovary demonstrates distinct functions for these two proteins.

## Discussion

The *Drosophila *genome encodes three homologues of the vertebrate SOCS. Each homologue contains the hallmark modular architecture, with a central SH2 domain followed by a carboxy-terminal SOCS domain. The genes are dispersed in the genome and are referred to by their cytological locations as *Socs16D*, *Socs36E*, and *Socs44A*. These fly SOCS genes are most similar to the vertebrate SOCS5, 6, and 7, none of which has been functionally characterized to date. Socs36E is the most similar in protein sequence to a vertebrate SOCS, SOCS5, but shares many characteristics with the extensively studied mammalian SOCS genes, SOCS1-3 and CIS. Each of these has been shown to be transcriptionally responsive to JAK pathway stimulation and act to downregulate JAK activity in a classical negative feedback loop [reviewed by [9]]. On the other hand, Socs44A is most similar to the less studied vertebrate genes, SOCS6 and 7. In this study, we demonstrated that Socs44A has properties that distinguish it from Socs36E and the canonical mammalian SOCS (compared in Table 3). First, the expression of *Socs44A *was not dependent on JAK pathway activity. Nevertheless, Socs44A was able to downregulate the JAK cascade in some, but not all tissues. In addition to regulating JAK pathway activity, *Socs44A *genetically interacts with the EGFR/MAPK pathway, acting to enhance its activity.

**Table 3:** Comparison of *Drosophila *SOCS.

	**Socs36E**	**Socs44A**
**Expression-Embryogenesis**	**Matches **known pattern of JAK activation, including pair-rule stripes, gut, and tracheal pits	**Distinct **from JAK activation, with possible exception of trachea very late
**Expression- Oogenesis**	**Matches **known pattern of JAK activation, with graded expression highest at anterior and posterior poles	**Distinct **from JAK activation, with expression only in nurse cells
**Requirement for expression **	**Requires **JAK signaling for embryonic expression	**Does not require **JAK signaling for embryonic expression
**Inducibility**	**Inducible **by JAK activity in embryos	**Not inducible **by JAK activity in embryos
**Regulation of JAK activity**	**Can repress **JAK signaling in wing and **possibly **in follicle cells of ovary	**Can repress **JAK signaling in wing, but **cannot **in follicle cells
**Regulation of EGFR activity**	**Can repress **EGFR signaling in wing and **possibly **in follicle cells of ovary	**Can enhance **EGFR signaling in wing

The contrasting properties of Socs36E and Socs44A are summarized.

### The *Drosophila *genome encodes three SOCS genes

Phylogenetically, SOCS fall into three general clades. The first includes the best studied vertebrate SOCS, CIS and SOCS1-3. Interestingly, there are no representatives of this group found in the fly genome. Vertebrate SOCS of the remaining two clades have yet to be fully characterized with regard to their physiological roles, as well as mechanistic roles in JAK/STAT signaling. *Socs36E *is most similar to the vertebrate SOCS of the second clade, containing SOCS4 and SOCS5. It shares similarity not only in the SH2 and SOCS domain, but also in the region upstream of the SH2 domain. Mutational analysis has shown that SOCS5 inhibits IL-6 [50], whereas nothing is known about the activity of SOCS4. *Socs44A *falls into the third clade occupied by vertebrate SOCS6 and SOCS7, as well as the only *C. elegans *homologue. SOCS6 has been shown to downregulate the insulin receptor [51,52]. Very little is known about SOCS7, other than its ability to interact with Nck, Ash, and PLCγ [53]. Because of the relative lack of information about these latter two clades, study of the *Drosophila *SOCS may identify general properties of these homologues that span each clade.

Although mammalian genomes encode large families of specific JAK pathway components, *Drosophila *has only one characterized receptor, *domeless*, one Janus kinase, *hop*, and a single STAT, *stat92E*. Despite the simplicity of the transduction machinery for the JAK pathway, there are three SOCS genes in flies. Furthermore, there is only one *Drosophila *homologue of the PIAS negative regulatory family, *zimp*, and it is also capable of inhibiting JAK pathway activity [54,55]. In an organism with few functionally redundant genes, why are there three *Drosophila *SOCS? Two possible explanations for the apparent abundance of SOCS are that the different *Drosophila *SOCS may be expressed differently or they may differently regulate signaling through pathways other than JAK. Indeed, we presented evidence for both of these distinctions for Socs36E and Socs44A.

### Socs44A does not participate in an auto-regulatory negative feedback loop

It has been demonstrated that, like the classical vertebrate SOCS genes, *Socs36E *is transcriptionally responsive to JAK pathway activity [[29] and this work]. In both embryos and ovaries, the expression of *Socs36E *mirrors the known pattern of JAK activation and, indeed, altered JAK activation in the embryo elicits a transcriptional alteration in *Socs36E*. Unlike *Socs36E*, the expression of *Socs44A *did not match that of JAK induction. In the embryo, detectable *Socs44A* expression was absent until late stages of embryogenesis, when it was restricted to the developing trachea. JAK activation does occur in the tracheal pits and has been implicated in tracheal morphogenesis [35,36], but *Socs44A *expression was lacking in the other tissues of the early embryo where JAK activation has been described. More telling was the finding that neither reduction nor expansion of JAK activation in the embryo had any effect on *Socs44A *expression. This disparity between *Socs44A *and *Socs36E *support the hypothesis that these genes are not redundant.

Despite the difference in expression of the two SOCS genes, both are able to downregulate JAK activity in some tissues. Misexpression of *Socs36E *is able to suppress JAK activity in the developing adult (imaginal) wing and thorax [28]. Similarly, misexpression of *Socs44A *reduced JAK activity in the imaginal wing, as illustrated by the enhancement of that phenotype by reduction of endogenous *hop*. Furthermore, misexpression of *Socs44A *rescued wing vein loss resulting from misexpression of *hop*. Perhaps most importantly, introduction of deficiencies that remove *Socs44A *rescued a *hop *wing vein phenotype. Taken together, these data strongly suggest that *Socs44A* downregulates JAK pathway activity during normal wing development. However, misexpression of *Socs44A *had no effect on expression of a marker for JAK pathway activity during oogenesis. This indicates that there is context specificity to SOCS action in *Drosophila*, a phenomenon that has been observed in the study of mammalian SOCS [43]. In contrast, misexpression of *Socs36E *was able to downregulate expression of the pnt-lacZ marker in follicle cells, although it cannot be distinguished whether this is due to reduction of signaling through JAK or EGFR. However, because *Socs36E *is expressed in the pattern of JAK activation in follicle cells, it is likely that it has a function in regulating JAK signaling in the ovary.

### Socs44A upregulates EGFR/MAPK signaling

Another distinction we noted between the *Drosophila *SOCS was in their abilities to regulate signal transduction cascades in addition to JAK/STAT. Precedence for such additional roles for vertebrate SOCS include regulation of Tec, Vav, TCR, c-kit, and FAK mediated signaling [56-60]. It has been previously shown that Socs36E can suppress signaling not only through the JAK pathway, but also through the EGFR/MAPK pathway [28]. Socs44A was also able to regulate EGFR/MAPK signaling, but acted in the opposite manner. Socs44A was able to rescue misexpression of the EGFR negative regulator *argos *in a dose-dependent manner. Furthermore, mutations in EGFR pathway components rescued Socs44A misexpression phenotypes. Importantly, a reduction of endogenous *Socs44A* activity enhanced the *argos *phenotype. Taken together, these data suggest that a normal function for Socs44A is to enhance the EGFR pathway. A potential mechanism for this genetic interaction can be found in a recent report describing physical interaction between SOCS3 and the p120 RasGAP [61]. p120 RasGAP, a GTPase-Activating Protein, is an antagonist of MAPK signaling that is responsible for inactivating Ras. It does so by stimulating Ras GTP hydrolytic activity, leaving Ras in a GDP-bound, inactive configuration. Upon interaction with SOCS3, p120 RasGAP is unable to inactivate Ras, resulting in an upregulation of the EGFR/MAPK pathway. Perhaps Socs44A is acting in an analogous manner. Indeed, there are three candidate RasGAP genes in the fly genome. Biochemical analyses will be required to address this hypothesis.

## Conclusions

There are three *Drosophila *SOCS, all of which have greatest homology to the two classes of vertebrate SOCS that are least well characterized. One of these, Socs36E, is a member of the vertebrate SOCS4/5 class and has been previously characterized [28,29]. It is similar to classical SOCS in that its expression is regulated by activity of the JAK pathway and that it functions to suppress JAK activity. Here we provided the initial characterization of Socs44A, a member of the vertebrate SOCS6/7 class. In contrast to *Socs36E*, activation of the JAK pathway was neither necessary nor sufficient for the expression of *Socs44A*. We conclude that Socs44A is unlike classical SOCS because it does not participate in a JAK pathway negative feedback loop. Still, Socs44A was capable of repressing JAK signaling, but that activity was limited to certain tissues. This context specificity is a feature that is shared with classical SOCS. Finally, Socs44A and Socs36E had opposite effects on EGFR/MAPK signaling. The enhancement of MAPK signaling that was seen for Socs44A is reminiscent of the influence of SOCS3 on this pathway, which is exerted through physical interaction of SOCS3 with p120 RasGAP. Perhaps a similar mechanism explains the enhancement of MAPK activity due to Socs44A. The differences observed here between Socs36E and Socs44A strongly suggest that they have distinct functions in the fly. Furthermore, the differences between Socs44A and the well studied class of canonical vertebrate SOCS may be representative of undiscovered distinctions amongst the three classes of vertebrate SOCS.

## Methods

### Comparison of SOCS sequences

Putative *Drosophila *SOCS genes were identified using a simple tBLASTn 2.0 query with a consensus sequence for the vertebrate SOCS domains [30] used to probe the complete genome contig sequences available from the BDGP. Identified homologies were compared with the predicted gene structures reported as "CG" sequences in the annotations of the genomic contigs. The translated sequences of the three putative SOCS gene genomic regions were scanned manually for possible alternative structures. The sequences surrounding the SOCS and SH2 domains were used to generate primers for the amplification of DNA corresponding to each putative gene. Amplification products were cloned and used to generate probes for the identification of cDNAs as described below. Phylogenetic comparison of SOCS proteins was performed using AlignX (VectorNTI 9.0), based on the ClustalW algorithm, to generate protein alignments and a neighbor-joining algorithm to create a phylogenetic tree.

### Identification of cDNAs

A cDNA library constructed from RNA of 12–24 hr old embryos [62] was screened using 800bp of genomic DNA derived from the 3' end of the *Socs36E *coding region, including the SOCS box and SH2 domain. Two independent clones (Genbank accessions AF435838 and AF435839) were recovered, with the former being structurally similar to an EST from the BDGP (clot #7147). The BDGP also recovered two cDNA clones representing *socs44A *which have been designated as clot #8463. We have determined the complete sequence of the longer clone, LP02169 (Genbank AF435923).

### In situ hybridizations

In situ hybridizations to embryos were performed as previously described [32]. Digoxigenin labeled probes for *Socs36E *and *Socs44A *were generated from the 5' ends of the respective cDNAs and did not include the coding region for the conserved SH2 and SOCS domains. Germline clone mutants for the *hop*^*c111 *^null allele were generated using the *ovo*^*D1 *^dominant female sterile technique [34]. Embryos derived from mutant mothers were collected overnight and prepared for hybridizations as previously indicated. Embryos misexpressing *upd *in a specific pattern were generated by crossing females carrying a UAS-upd transgene with males heterozygous for paired-GAL4, which expresses GAL4 in the seven stripe pair-rule pattern of the *paired *gene. Progeny were collected and hybridized as above. Trachea in germline clone-derived *hop*^*c111 *^embryos were visualized with the *trh*^*10512 *^enhancer trap [63] using anti-β-gal antibody (Cortex Biochemical, at 1:1000) as previously described [26].

### Misexpression studies

To express *Socs36E *and *Socs44A *under control of GAL4, the full-length cDNAs described above were cloned into the pUAST vector [64]. Germline transformations were performed [65] and transgenic lines established. For wing phenotypes, *engrailed*-GAL4 (e16E-GAL) was used to drive expression of the transgenes in the posterior compartment. Wings were dissected and mounted in Hoyer's medium [66] for photography.

Ovarian clones of the null allele, *hop*^*c111*^, were generated by hsFLP mediated mitotic recombination as previously described [26,33]. Misexpression clones of *Socs36E *and *Socs44A *were generated using a GAL4 flip-out cassette [67]. Genotypes of those animals were *w [hsFLP]1*; *[Act5C>y>GAL4] [UAS-GFP.S65T]/ [UAS-socs36E]11.2 *and *w [hsFLP]1*; *[Act5C>y>GAL4] [UAS-GFP.S65T]/*+; *[UAS-socs44A]/ pnt-LacZ*, respectively. For each, ovaries were fixed and stained with anti-β gal and anti-GFP as previously described [26,33].

### Microscopy

All *in situ *hybridization and wing images were acquired using a Spot Camera (Diagnostic Instruments) on a Nikon E800 microscope using differential interference contrast (DIC). A Leica TCS-SP laser scanning confocal microscope was used to capture all fluorescence micrographs. All images were then exported to Adobe Photoshop for manipulation and annotation.

## Authors' contributions

JR performed experiments with Socs44A and participated in drafting the manuscript. GR performed most experiments with Socs36E. SH performed some experiments with Socs36E. RX performed immunofluorescence experiments in ovaries, except for Socs44A. DH conceived of the study, participated in its design and coordination, and participated in drafting the manuscript. All authors read and approved the final manuscript.
